# Functional Biscuits, a Healthy Addition to Your Coffee Break—Evaluating Consumer Acceptability and Willingness to Pay

**DOI:** 10.3390/foods13111731

**Published:** 2024-05-31

**Authors:** Emanuele Blasi, Eleonora Sofia Rossi, Roberta Pietrangeli, Marco Nasso, Clara Cicatiello, Samuela Palombieri, Francesco Sestili

**Affiliations:** 1 Department for Innovation in Biological, Agro-Food and Forest Systems (DIBAF), University of Tuscia, Via San Camillo del Lellis snc, 01100 Viterbo, Italy; e.blasi@unitus.it (E.B.); marco.nasso@unitus.it (M.N.); cicatiello@unitus.it (C.C.); 2 Department of Economics, Engineering, Society and Business Organization (DEIM), University of Tuscia, Via del Paradiso 47, 01100 Viterbo, Italy; roberta.pietrangeli@unitus.it; 3 Department of Agriculture and Forest Sciences (DAFNE), Via San Camillo de Lellis, 01100 Viterbo, Italy; palombieri@unitus.it (S.P.); francescosestili@unitus.it (F.S.)

**Keywords:** sensory perception, finite mixture model, health claim, high-amylose bread wheat, snacking, diet and nutrition

## Abstract

An increasing number of individuals are eating out due to work and study commitments. This trend directly influences people’s food choices, especially those who frequently rely on snacks and pre-packaged foods. Consuming these foods can lead to long-term health consequences. Adding functional foods to vending machines could lead to healthier choices. Our aim is to evaluate the acceptability and willingness to pay (WTP) of workers and students for a snack pack of novel functional biscuits (FBs) made with high amylose contents. We found that the experimental flour used is effective in preventing various non-communicable diseases; two phases of analysis were carried out on 209 participants. The participants blindly tested the products and only after the sensory evaluation were they informed about the biscuits’ health contents. Firstly, the blind investigation highlighted the acceptability of the FBs compared to the conventional biscuits. Secondly, the finite mixture model on WTP revealed that some consumers are interested in the health benefits associated with high-amylose test blends and others are focused on hedonistic taste. The design of a communication strategy and industry approach should aim to assist consumers in comprehending the health benefits and sensory aspects of novel functional foods available on the market.

## 1. Introduction

Eating patterns are changing significantly in the modern era [1]. An increasing number of people are regularly eating out because of work and study commitments, which has a direct impact on their food choices [2,3,4]. For convenience and efficiency, snacks and pre-packaged foods are often the choice for a short lunch or a break [5,6,7]. Typically, the snacks available at vending machines lack nutritional value and are rich in salt or sugar and low in essential nutrients. Consuming these foods can lead to an increased intake of calories, fats, and sugars, potentially resulting in adverse health effects over time [8,9,10]. Obesity, diabetes, cardiovascular issues, and a decrease in energy and concentration are linked to poor eating habits and sedentary lifestyles [11,12,13,14,15]. This trend has generated greater awareness about the importance of proper nutrition in maintaining good health mainly because of educational campaigns promoted by governments and associations, particularly in high-income countries [16].

Functional foods (FFs) are becoming more popular among consumers who are concerned about their physical well-being and looking for healthy foods on the market [17,18]. Some of the ingredients in these products have been demonstrated to improve health or provide additional benefits beyond those offered by conventional foods [19,20]. Due to the growing demand, the food industry is investing in research and development to create functional food products that have positive physiological benefits while maintaining the same shape and taste as common food products [16,21]. There is evidence that sensory aspects can be barriers to the acceptance of FF by consumers [22,23,24]. The consumer interest in these products can also be influenced by the individual characteristics of consumers including gender, age, and eating habits [25,26]. Several studies explored the FF acceptance also considering conveyed informative content [25,27,28,29]. Functional content is an intangible attribute for the consumer that cannot be immediately verified [30,31]. Therefore, market designers employ a variety of methods to convey information. By separating Nutrition Claims (NCs) from Health Claims (HCs) on the product label, it becomes simpler to identify benefits related to the message that is being communicated. HC and NC-labelled foods are perceived as healthier by consumers, and they are willing to pay more for them [22,32,33,34]. Claims that describe potential health benefits, such as reducing the risk of chronic or degenerative diseases, can help increase the willingness to pay (WTP) for FF [25,29,35].

Based on these premises, this study examines a functional snack made with a new experimental flour that is meant to prevent non-communicable diseases (NCDs). The goal of this study is to expand the literature on the subject, specifically targeting workers’ and students’ potential interest in this type of FF that could assist people who eat out in adopting healthier eating habits while on breaks without affecting taste. The focus of our study is on biscuits with a high content of amylose wheat flour, packaged as a snack of four biscuits, fitting in vending machines located in study rooms or workplaces.

Amylose content is associated with resistant starch content in foods, which plays a role similar to dietary fibre in preventing several NCDs, such as type 2 diabetes, obesity, colon cancer, and cardiovascular disorders [36]. Starch is the main component of wheat endosperm (80% dry matter) and is composed of approximately 20–25% amylose and 75–80% amylopectin. Through the manipulation of a starch biosynthetic gene, a new bread wheat line (*Triticum aestivum*), Cadenza SBEIIa*, with a high amylose content (contents greater than 70%) has been developed [37]. The derived flour and foods have showed a positive impact on human health by acting on NCD prevention [38,39,40]. Scientific evidence suggests that high-amylose biscuits offer better glycaemic control compared to the control counterparts [39,41]. Dell’Unto et al. [42] conducted an online survey that revealed that consumers present a potential interest in high-amylose bakery products, expressed in premium price recognition.

Starting from this insight, the goal of the present research is twofold: 

(1) testing the acceptability in different information contexts through the development of sensory evaluations and (2) estimate the potential WTP, also considering the HC conveyed on the snack pack of high-amylose biscuits. Moreover, a finite mixture model was applied to investigate the potential different types of consumers in our sample to provide interesting insights for industrial and marketing improvement.

## 2. Materials and Methods

### 2.1. Experimental Design

Tests were performed to examine consumers’ acceptability and WTP for baked goods, such as biscuits, using flour mixtures with high amylose contents. According to the approval waived by the Ethics Committee of University of Tuscia, the participants blindly tested the products and only after the taste evaluation were they informed of the health contents of the biscuits. Informed consent was obtained from all subjects involved in the study.

Sensory studies can be utilized to collect information about market acceptance of substituting ingredients or processing new food products [43,44,45], while tasting tests can be used to investigate the perception of different sensory attributes [46,47,48]. In the literature, the RATA (Rate-All-That-Apply) methodology has been recognized as a valid technique for gathering more information and increasing the discriminative capacity relating to the sensory attributes of products [49]. It represents a variant of the CATA (Check-All-That-Apply) technique [50], which allows us to link the attribute evaluations to an intensity scale. RATA can be utilized to measure the presence, absence, and intensity scale of food attributes that are not yet on the market [47,51,52,53].

In the present research, the following three categories of items related to bakery products were identified and tested in accordance with Delicato et al.’s [54] approach: texture, taste, and appearance. Based on the results from the pilot test on 10 respondents [39], the analysis involved 14 attributes that were divided into relevant categories. 

### 2.2. Raw Material, and Product Presentation

**Figure 1:**
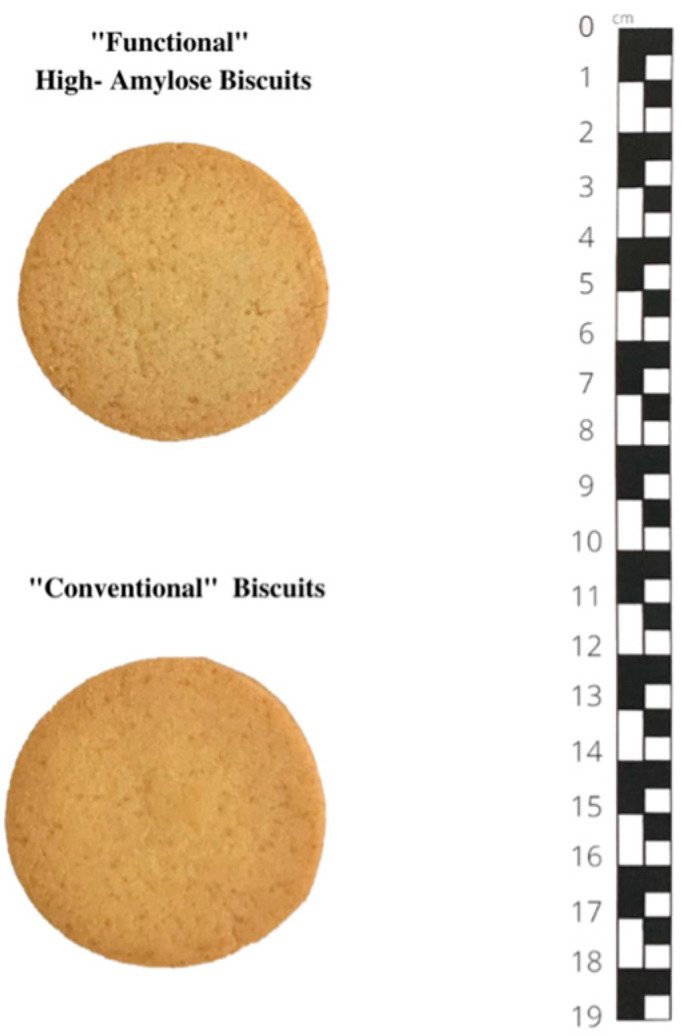
Functional “high-amylose” and conventional biscuits.

Two biscuits were prepared using distinct flour blends to test acceptability and sensory evaluation for the taste test. Functional biscuits (FBs) and conventional biscuits (CBs) had the same shape, size, and colour (Figure 1).

The experimental and conventional flours were used to make the biscuits, one enriched with amylose and resistant starch and the other with 65% commercial type 1 flour and 35% type 00 flour, to achieve the same structural chemical composition as the experimental flour. The high amylose content flour (75% vs. 30% of the conventional flour) was the novel ingredient used to make the sample test of the functional biscuits. The products were prepared in a professional bakery according to the procedures and equipment used by Di Rosa et al. [39]. The recipes differ only in the flour typology (Table 1).

**Figure 2:**
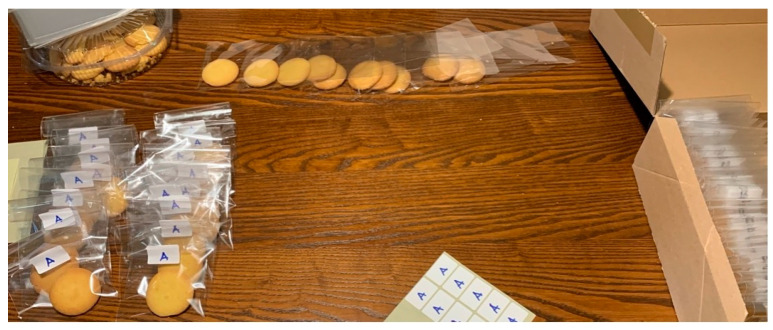
Material preparation.

Each participant received a tasting sample with one biscuit per type (Figure 2). The food packets were identical and transparent with the same amount of food (g) and only two letters (A and B) were marked to identify the taste order. Furthermore, the participants received a glass of water to avoid any influence on the tastings. The purpose of this was to clean the mouth between two tastes.

### 2.3. Participants

The 209 participants were recruited among students, university staff, professors and researchers and participants in an event held by the university between May and June 2022. The two sessions, on two different days, were carried out in front of two buildings of the University of Tuscia between 9.30 a.m. and 12.30 a.m. The research was carried out outdoors in compliance with COVID-19 restrictions. The study involved people of an age range between 18 and 66 years old. The focus of this study was on individuals eating away from home for work or study purposes, including both main meals and breaks. These individuals might not be attentive to the health implications of the food they consume outside of their home [6,8].

Participants with food allergies/intolerances (e.g., lactose, eggs, and gluten) were excluded from the study.

### 2.4. Experimental Procedures

**Figure 3:**
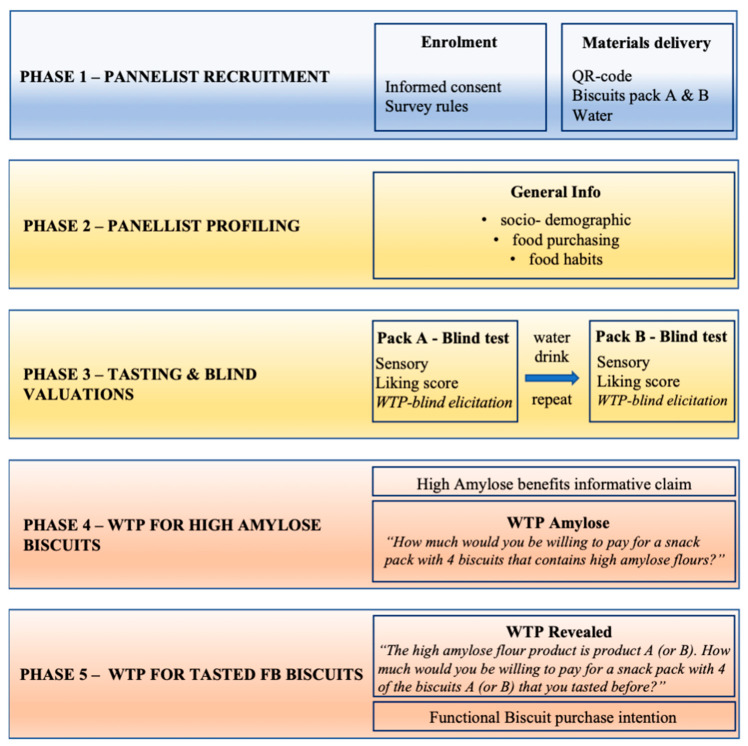
Experiment phases.

**Figure 4:**
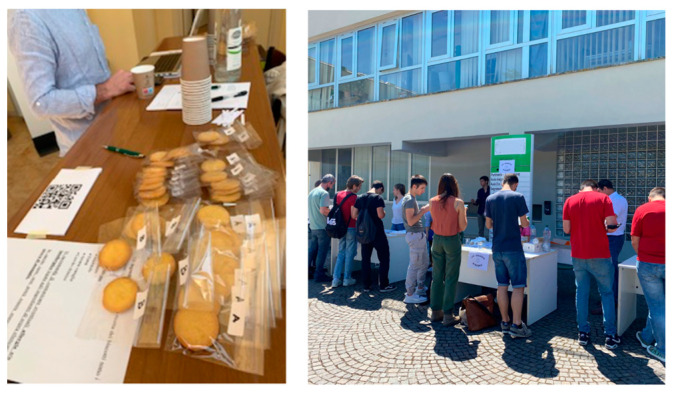
Workstation arrangement.

The experimental procedure included five phases (see Figure 3), which are described in detail below:

To welcome participants and collect the necessary materials for the survey, two identical workstations were set up with the same number and arrangement of products (Figure 4). To avoid bias, the participants were not informed about this difference.

The survey began after participants filled out the informed consent form following the Declaration of Helsinki guidelines and received a unique code. This code was only used to prevent the same person from answering the questionnaire multiple times, not to identify the interviewees. 

By scanning the QR code on the workstation, the questionnaire was accessed using a personal smartphone.

#### Questionnaire

Google Forms was used to create the survey, which consisted of two questionnaires, A and B. The two biscuits were tasted in opposite order for each subgroup. In questionnaire A, the ‘functional’ biscuit was the first one to be tasted (A), while in group B, the biscuit with high amylose was tasted second (marked with letter B). All participants rated two biscuits with an ever-increasing degree of information. The analyses involved both sensorial tasting and WTP elicitation through open/closed questions at the different information levels provided.

The questionnaire included 6 sections as shown in Table 2 below:

The 1–9 Likert scale was used in Section 2 to measure the attributes that can impact food purchasing habits (1—not at all; 9—very often). Participants’ familiarity and consumption of snacks were measured by examining the frequency of purchases and average price of snacks purchased from vending machines (Section 3). The two biscuits were blindly tested by the respondents in Section 4. The sensory evaluation is based on the 1–9 Likert scale (1—not at all; 9—very often) according to RATA methodology. 

The WTP for a snack pack of 4 biscuits was measured using an open question in the following different informational situations:

- WTP BLIND: the blind test involved the participants estimating their WTP for each of the two biscuits tasted without any information about the product. (Phase 3—Section 4): “How much would you be willing to pay for a snack pack with 4 of these biscuits you just tasted?”
- WTP AMYLOSE: respondents declared their WTP after the health claim informed them on the properties of high amylose but did not mention any relationship with the tasted biscuits. (Phase 4—Section 5): “How much would you be willing to pay for a snack pack with 4 biscuits that contains high amylose flour?”
- WTP REVEALED (WTP-R): after identifying which biscuit contained amylose, the WTP of the respondent was identified. (Phase 5—Section 6): “The high amylose flour biscuit is product X. How much would you be WTP for a snack pack with 4 of the biscuits X that you tasted before?”

### 2.5. Data Analysis

#### 2.5.1. Sample Acceptability of Functional Biscuits

Blind test data were utilized to evaluate the acceptability of functional biscuits by measuring sensory attributes, liking level, and WTP. Paired t-tests were used to compare these biscuits to the conventional biscuits [55,56,57].

#### 2.5.2. Heterogeneity Analysis

Functional product acceptance and WTP can be influenced by variables such as purchasing habits and individual characteristics [17,27,28,32,58,59].

As suggested by Teuber et al. [60], our research considers the frequency of purchasing a snack as a proxy of familiarity with the investigated product. In this analysis, four levels of purchase frequency of snack packs of biscuits were considered (1: very rarely; 4: very often). 

As our research is on functional foods, the variables also included purchasing habits such as caloric content and healthiness, developed using a Likert scale (1: not at all; 9: very often). For the variables examined, the presence of multicollinearity was excluded. The correlation matrix showed that none of the selected items had a correlation index higher than 0.65. 

The finite mixture model (FMM) was selected to examine the potential influence of these factors on consumer preferences and WTP. Marketing literature frequently employs mixture models to target consumers [61,62,63,64,65]. 

The FMM approach can simultaneously calculate the clustering process and OLS regression estimation. Using FMM, it is possible to divide the sample into different consumers groups by investigating the factors that affect WTP for functional biscuits. In the analyses, socio-demographic characteristics, familiarity and average purchase price were used as grouping factors. Furthermore, among the socio-demographics, the student dummy (1 = students, 0 = otherwise) was considered a work category. The WTP REVEALED of functional biscuits was analyzed by considering purchasing habits, FB liking level and WTPInfoAMYLOSE (WTPiA).

To assess the interest in amylose HC, WTP amylose was used, but by eliminating the potential anchoring effect that the WTP BLIND could generate. The WTPinfoAMYLOSE (WTPiA) variable was calculated as follows:

WTPinfoAMYLOSE*_i_* = WTP*_i amylose_* − ΔWTP*_i blind_* (1)

where ΔWTP*_i blind_* was calculated between the WTP BLIND for the CB and FB, considering that the *t*-tests did not reveal significant differences.

The FMM estimated a regression for each of the sample data clusters. The expectation–maximization algorithm, defined by Dempster et al. [66], estimates the coefficients of each class without prior information on clustering. The function of *wtp_i_* is the set of personal factors *x*_1_ of the *i*-th individual characterized by *x*_2_ socio-demographic characteristics in a sample of size *n*. The objective function of maximum likelihood relating wtp*_i_* and x_1_ is as follows:

Π*_n_*π*_ij_f*(wtp*_i_*|x*_i_*_1_, z*_i_* = 1) (2)

where *π_ij_* is the probability of observation *i* = 1,…, *n* that belongs to a given group *j* = 1, …, *K*, where *K* represents the number of groups that are endogenously determined in the model, and *z_i_* is a non-observable latent variable that determines if an observation is involved in one group or another. The likelihood combines the conditional likelihood of each latent class weighted by the associated latent-class probability. For each observation, the probability of inclusion in group *j*, *π_ij_*, is a function of *z_i_*, which assumes values 0 or 1 to define the exclusion or inclusion of the ith observation in the specific *j*th group as follows:

π*_ij_* _=_ π (z*_i_* = 1) = exp(z*_i_*)/Ʃ*_j_*_= 1,*K*_ exp (z*_i_*) (3)

Equation (3) yields the expectation step of the algorithm.

Given that it is unknown which group a certain observation comes from, the observed sample is incomplete since *z_i_* is a latent and non-observable variable. z*_i_* can be approximated by the set of variables *x*_2*i*_. A multinomial logit model computes *π_ij_* as follows:

π*_ij_* = h(x*_ij_*) = exp (g(x*_i2_*))/Ʃ*_j_*_= 1,*K*_ exp (g(x*_j_*_2_)) (4)

where *g*(*x_i_*_2_) is the function relating the probability of being in the *j* class to the *x_i_*_1_ characteristics.

The linear model explains the dependent variable wtp_i_ as a function of the factors in *x_i_*_1_. The expectation–maximization algorithm allows the contemporaneous estimation of the probability to belong to a group *π_ij_* together with the regression coefficients *β_j_* in each of the *K* groups, breaking the objective function iterating between the estimated expected probability of belonging to a specific group, *E*(*π_ij_*) in (4), and the estimates of the regression coefficients for each group (*β_j_*) in (2). STATA15.1 software was utilized for the estimation of all models (StataCorp LP, College Station, TX, USA).

## 3. Results

### 3.1. Sample Description

Table 3 presents the main socio-economic characteristics of the interviewees. The sample is quite young because more than 70% are under 35 years old; the average age is about 30 years old. Gender is evenly distributed in the sample, with 44.50% being women. The percentage of respondents with a household size of four people is 44%, while only 9% are made up of a single person. Additionally, approximately 60% of the interviewees are students and around 30% are employees. More than 40% of respondents purchase snack packs from vending machines frequently (often/very often). Respondents prioritize healthiness (7.91), price (6.95), and certification presence (6.75) over brand (5.19) during their food purchasing processes (expressed in Likert scale 1–9).

### 3.2. Acceptability of Functional Biscuits (WTP, Liking, Preference and Sensory Profiling) 

Unpaired two-sample *t*-tests (UTS-test) between subgroups A (first FB) and B (second FB) show no statistically significant differences in taste order. The two subgroups were treated as one sample of 209 individuals. Table 4 shows the paired *t*-test estimates for sensory attributes, liking level and WTP of functional biscuits compared to conventional ones. The sensory attributes are categorized into three macro-categories: appearance, texture, and taste. In only four attributes, the blind-condition test phase revealed significant differences. As indicated by the analyses of the attributes crumbly (*p*< 0.0153), chewy (*p* < 0.0001), crisp (*p* < 0.0021) and pasty (*p* < 0.0001), the CB presents a greater sensation of friability and crunchiness. These aspects appeared to have no impact on the general level of acceptability of FB. Paired t-tests performed on the liking level and WTP did not reveal any statistically significant differences. Without any product information, it can be concluded that the interviewees showed general acceptability of both functional and conventional biscuits.

### 3.3. Heterogeneity Analysis—Finite Mixture Model Regression Results

Akaike’s information criterion (AIC) and Bayesian information criterion (BIC) were used to determine the optimal number of classes, shown as *K* [61]. The best fit is identified in the subdivision of the sample into three classes (*K* = 3). The Bonferroni ANOVA test was carried out to confirm the statistical significance of the difference in the means of the estimated groups. The description of the groups is defined at the significance level of *p* < 0.05. Table 5 shows the FMM model estimation. It provides a description of both the factors that determine the probability of belonging to each group (π) and the role of various factors in the WTP REVEALED within each group. Table 6 shows the average value of the main variables for each group. 

Table 5 shows the probability of group membership. The estimate characteristics are based on the reference group, which in our case is Group 1. The estimates reveal that individuals in Group 2 pay more for biscuit packs than the reference group. Group 3 consists predominantly of women who purchase snack packs less frequently but at a higher price than Group 1. The OLS estimates (Table 5) for the WTP REVEALED on FB provide interesting insights from the group analysis. According to our results, the FB liking level in all three groups has a positive impact on the WTP-R, which results in an increase in price from EUR 0.10 to EUR 0.20 per pack. The WTPinfoAMYLOSE (proxy for amylose HC interest) is a significant factor in influencing WTP-R.

Variables such as caloric contents, FB liking, and WTPiA play a role in affecting the WTP revealed in Group 1 (42% of the sample). According to Table 6, this group is the youngest (around 28 years old) and mostly consists of students (70%) and males (60%). It also has the lowest average WTP-R, around EUR 0.83 per pack. This group appears to prefer price and certification presence in their purchasing habits. Group 1 occasionally buys snacks in packs and spends a smaller budget than the other ones (EUR 0.99). The HC of amylose is valued on average at EUR 0.32 per pack, very close to the value declared by Group 2. We can define Group 1 as “The budget-conscious” group.

Table 5 indicates that purchasing habits, such as attention towards certified products and price, have a positive effect on the WTP-R of Group 2 (43% of the interviewees). Moreover, this group indicates the highest increase in WTP REVEALED, considering the WTPiA. In fact, by increasing the WTPinfoAMYLOSE to EUR 1, the WTP-R increases by almost the same amount (EUR 0.90). Group 2’s statistics (Table 6) show that men and women are evenly divided, with the highest average age (33 years old). About EUR 1.40 per pack is the value declared by the components for the WTP REVEALED. Hence, this group frequently buys snack packs with an average price of EUR 1.15 and, as a result, is known as “*The snack-addicted*” group. 

In Group 3 (15% of the sample), various factors influence the WTP REVEALED (Table 5). Our observation regarding purchasing habits shows that paying attention to healthy food and the food brand decreases the WTP-R (EUR 0.50 and EUR 0.13 per pack, respectively), but paying attention to certification (EUR 0.23), calorie content (EUR 0.10) and price (EUR 0.13) increases the WTP-R. According to Table 6, this group, which is the most satisfied in terms of FB liking level, has the highest correlation between the approval of the functional biscuit and the WTP-R with an increase of EUR 0.20 per point. The group is composed of 60% women, with an average age of around 30 years. Despite paying EUR 1.35 per pack (the highest value), they rarely purchase snacks. This group has the highest WTP-R value (around EUR 2.40 per pack). Group 3 is identified as “*The health-focused*” group due to the emphasis on health. It can be deduced from the healthiness and calorie content values of food purchasing habits and from the WTPiA value, which is the highest (approximately EUR 0.50 per pack).

## 4. Discussion

This exploratory study is designed to gain insight into the acceptance of a functional snack made with high amylose content flour and how various factors influence the willingness to pay for them by various consumer groups.

The blind tasting test was fundamental to understand any sensory differences between the two biscuits, without potential biases that could be due to personal information or attitudes. Empirical evidence from the research supports the acceptability of novel functional biscuits among the sample of consumers. Respondents observed slight distinctions in sensory aspects between FB and CB during the blind tasting, including chewiness, crumbliness, crunchiness, and pastiness. Amylose and starch’s ability to absorb water is what drives these differences [40,67]. FB biscuits are significantly higher in amylose and starch compared to conventional ones. According to a study by Di Rosa et al. [39], biscuits with a high level of amylose contain more resistant starch, approximately 10 times more than the conventional biscuits. Despite the texture differences between the two biscuits, the tasting test revealed that it did not affect the overall liking level. As Delicato et al. [54] suggest, it is feasible to substitute certain levels of conventional ingredients with functional ones in baked goods, such as biscuits, without affecting consumer acceptance. Furthermore, the declaration of willingness to pay between the two biscuits without any additional information did not show significant differences. Once the health claim about the FB nutritional content was communicated, the differences were observed.

This leads us to the second topic of our analysis. According to the heterogeneity analysis results, the sensory acceptability of a biscuit is connected to the functional content’s healthiness, resulting in a greater WTP. The FMM allowed the sample to be divided into sub-groups of consumers. The product being investigated in this research is a biscuit, a daily food product that has a strong hedonic value for consumers. Using this approach, other studies have investigated the market segmentation of products that have a strong prevalence in the hedonic sphere, such as wine [62,63,64]. Bertail and Caillavet [61] instead use the FMM to analyze consumers of everyday food products such as fruits and vegetables.

Our findings suggest that health claims about high levels of amylose and taste-experienced content may have a positive impact on WTP. This is aligned with the outcomes obtained by Lawless et al. [68], who observed a higher WTP when the consumer tastes the product and then receives the health-related information. The satisfaction of the experiential content, expressed as the FB liking level, seems to be linked to a greater propensity to spend more, confirming the claims of the reference literature, including work by Verbeke (2006). This highlights the importance of including novel FF aspects connected to taste evaluation in WTP analysis. Furthermore, combining the experiential content with the amylose HC is considered positively as “new taste knowledge” of the health benefits provided by functional biscuits. Consumers become more inclined to spend more on a tasty but also healthy product after learning which biscuit is functional. This result is consistent with other studies stating that consumers recognize a greater WTP for products with clear indications related to sustainability aspects [69,70,71] or to HC for functional products [25,29,42,72,73]. Our sample’s purchasing habits may have a different impact on their WTP, according to the FMM results. “The budget-conscious” group is represented by young students who, during their purchasing habits, pay attention to price. In the case of purchases with a limited budget, their behaviour leads them to search for better quality, as indicated by the attention towards certifications, which are recognized as having an essential role in the transmission of information and validation of credence attributes [35,74,75,76,77]. Looking at WTPinfoAMYLOSE, our insights seem to demonstrate an evolution over time of young consumers who are more aware of the effects that a balanced diet can have on their health, contrary to what was analyzed, e.g., [26,78,79]. However, their WTP-R for functional biscuits remains the lowest among the different groups, and it is probably even lower than the market price for conventional snacks. “The snack-addicted” group represents the consumers most familiar with snacks and their WTP is also strongly influenced by HC. Similar results were provided, for example, by Teuber et al. [60], who suggested that consumers declared higher WTP when they are more confident with the product and health claims. We should consider that specific food categories, such as biscuits, are primarily considered hedonistic by a segment of consumers, as demonstrated by studies by Dean et al. [80], Papoutsi et al. [23], and VanKleef et al. [72]. Considering that snacks from vending machines during study/work breaks are commonly chosen for their hedonistic contents, these findings are worth highlighting [8,12,81]. Based on our results, this group of consumers may be attracted to this type of food that improves their health while still maintaining their expected hedonistic qualities. The majority of female participants in “The health-focused” group have validated that women are more attentive to the health aspects of HCs on food products. Previous studies, such as [32,73,82], have documented similar findings. Furthermore, research on snacks has demonstrated that women consume snacks less frequently than men and choose healthier alternatives, which supports the attitudes of this segment of the sample [2,83,84,85].

The study emphasizes that our sample’s consumers are interested in functional products that they commonly consume and have a hedonic value that should not be ignored. This supports the findings of Ares and Gámbaro [86] that FF development should be tailored to age groups and consumer preferences. The positive result on the acceptance of innovative blends with high amylose content is an encouraging result for the food industry to overcome the barriers of taste that have often hindered the choice of FFs by the market [23,28]. Functional food appreciation can grow by consumers thanks to the evolution of food technology and the improvement of recipes. To ensure that consumers perceive that the taste of a biscuit has been preserved, it is crucial to emphasize this aspect in dedicated communication plans. Moreover, the other groups in our survey seem to be interested in the health benefits associated with high-amylose experimental flour blends. The importance of indicating the correct health claim to consumers cannot be overstated or understated [87]. A communication strategy is essential to ensure clear and impactful messages on the essential information identified from our analysis. This strategy should also be designed to validate the credence attributes of this novel product, such as functional content, hedonistic content, and taste, which can only be confirmed after purchase.

## 5. Conclusions

This research highlights that functional high-amylose biscuits are accepted despite some sensory differences, but there are different consumer groups that are affected by different factors in their WTP. The analysis revealed the importance of maintaining the hedonic content and taste even in a functional snack and conveying the correct information. This study confirms that it is essential to accurately identify target consumers who can appreciate the product and are willing to pay more for it. Our sample’s interest in this novel product, even though it is not representative of the entire population due to non-probability sampling, is a significant insight. Vending machines could offer a healthier option, without compromising on the hedonistic pleasure, for those who prefer sweet snacks during study and/or work breaks. This snack can help prevent several non-communicable diseases that are often associated with their lifestyle such as type 2 diabetes, obesity, colon cancer, and cardiovascular disorders. 

It is important to note some significant limitations in our paper. In addition to the non-representative nature of the sample, our analysis is intended as a preliminary analysis on high-amylose experimental flour. The objective of presenting the product for blind tasting and testing the potential HC to be delivered to consumers was achieved by using anonymous packaging. Future research must examine packaging with tested claims. Moreover, the analysis did not foresee any real market consequences that could therefore have led to bias of overestimation of the declared WTP values. The WTP estimates may be considered as plausible trends rather than precise numbers. Considering the limitations of this study, future empirical work could also expand the analysis on high-amylose products. It would be interesting to understand if this type of product is also accepted at different ages such as adolescence and pre-adolescence and in other categories of workers with a sedentary lifestyle. Furthermore, high-amylose flour is suitable for further testing of other bakery products. By analysing responses for various types of products and age groups of consumers, it is possible to assess and tailor products to different target consumers.

## Figures and Tables

**Table 1:** Ingredients and quantities used in the recipes of the biscuits’ formulations tested in the study.

Ingredients	Functional High-Amylose Biscuits	Conventional Biscuits
High-amylose wheat flour (g)	2000	--
“Conventional” wheat flour (g)	--	2000
Butter (g)	1050	1050
Salt (g)	10	10
White sugar (g)	400	400
Eggs (g)	280	280
Vanilla extract (g)	4	4
Maltitol (g)	300	300

**Table 2:** Questionnaire sections.

Questionnaire Sections	Information
1 Socio-demographic information	Age, gender, household size, work categories
2 Food purchasing processes	Healthiness, certification, brand, price, calorie content, quality, brand, packaging
3 Snack pack purchasing	Purchasing frequency, price
4.a Blind biscuit condition evaluation—biscuit I	Sensory attributes, liking level, WTP
4.b Blind biscuit condition evaluation—biscuit II	Sensory attributes, liking level, WTP
5 Amylose information	WTP
6 Revealed functional biscuit	WTP, purchasing intention

**Table 3:** Descriptive and summary statistics of the variables (Obs 209).

Variables	Sample
Socio-demographic variables	
Age (mean)	30.55
18–25 years (%)	43.06
26–35 years (%)	32.54
36–45 years (%)	11.00
46–55 years (%)	6.70
>55 years (%)	6.70
Gender (women) (%)	44.50
Household size (%)	
1	9.09
2	13.40
3	18.66
4	44.50
5 or more	14.35
Work categories (%)	
Freelance	2.87
Employee (public/private)	29.19
Students	61.24
Retired	0.96
Other	5.74
Attitudinal variables	
Snack-pack purchase frequency (%)	
Very rarely	22.97
Rarely	44.02
Often	30.62
Very often	2.39
Purchasing habits ^1^	
Healthiness	7.91
Certification presence	6.75
Calorie contents	6.18
Price	6.95
Brand	5.19

^1^ Data are expressed as average scores on Likert scale 1–9 (1: not at all–9: very often).

**Table 4:** The *t*-test acceptability variables under blind conditions.

Macro Categories	Variables	Functional High-Amylose Biscuits	Conventional Biscuits	Paired *t*-Test (*p*-Value)
General acceptability				
	WTP ^1^	1.03 (0.59)	1.04 (0.60)	0.2840
	Liking level ^2^	6.98 (1.53)	7.05 (1.37)	0.5309
Sensory attributes ^2^				
Appearance	Big	5.22 (1.58)	5.25 (1.62)	0.8294
	Small	4.39 (1.76)	4.54 (1.95)	0.1578
	Thick	4.38 (1.75)	4.23 (1.79)	0.1529
	Thin	5.31 (2.00)	5.36 (1.99)	0.7159
Texture	Crumbly	6.66 (1.59)	6.97 (1.56)	0.0153
	Chewy	2.74 (1.93)	2.25 (1.57)	0.0001
	Crisp	5.21 (2.02)	5.69 (1.88)	0.0021
	Pasty	4.97 (2.16)	4.38 (2.24)	0.0001
	Dry	4.53 (2.06)	4.62 (2.15)	0.5444
Taste	Fat	5.38 (2.01)	5.59 (2.14)	0.1587
	Savory	2.70 (1.67)	2.87 (1.85)	0.1792
	Sweet	6.33 (1.51)	6.42 (1.47)	0.4140
	Toasted	4.39 (2.17)	4.82 (2.06)	0.0051
	Bitter	1.66 (1.10)	1.90 (1.53)	0.0207

Obs = 209; standard deviation in parentheses. ^1^ Data are expressed as average scores EUR/snack pack of 4 biscuits. ^2^ Data are expressed as average scores on Likert scale 1–9 (1: not at all–9: very often).

**Table 5:** Finite mixture model results (own elaborations).

Variables	Group 1 “The Budget-Conscious”		Group 2 “The Snack-Addicted”		Group 3 “The Health-Focused”	
WTP REVEALED estimates within each group and their probabilities						
	Coeff. (std. errs.)	*p*-value	Coeff. (std. errs.)	*p*-value	Coeff. (std. errs.)	*p*-value
Healthiness	0.01(0.03)	0.744	−0.04 (0.04)	0.301	−0.49 (0.08)	0.000
Certification presence	0.02 (0.02)	0.204	0.05 (0.02)	0.042	0.23 (0.05)	0.000
Calorie content	0.03 (0.01)	0.023	−0.02 (0.02)	0.302	0.10 (0.04)	0.029
Price	0.03 (0.02)	0.111	0.07 (0.03)	0.042	0.13 (0.05)	0.015
Brand	0.01 (0.02)	0.588	−0.01(0.02)	0.481	−0.13 (0.03)	0.000
FB liking level	0.08 (0.02)	0.000	0.11 (0.02)	0.000	0.18 (0.05)	0.000
WTPinfoAMYLOSE	0.71 (0.05)	0.000	0.90 (0.04)	0.000	0.87 (0.12)	0.000
Constant	−0.63 (0.23)	0.006	−0.01 (0.32)	0.982	2.30 (0.84)	0.006
Factors estimates determining the probability of belonging to each group						
Gender ^1^	0		0.52 (0.45)	0.241	0.96 (0.52)	0.067
Age	0		0.04 (0.03)	0.196	0.01 (0.04)	0.763
Student ^2^	0		−0.21 (0.61)	0.729	−0.50 (0.76)	0.511
Snack purchase frequency	0		−0.31 (0.33)	0.347	−1.05 (0.40)	0.009
Purchase price of snack	0		1.52 (0.65)	0.019	3.32 (0.81)	0.000
Constant	0		−2.16 (1.59)	0.176	−3.15 (1.82)	0.083
π	0.42 (0.07)		0.43 (0.07)		0.15 (0.03)	
Log likelihood = −119.68737						

Obs = 209; standard deviation in parentheses. ^1^ (dummy 1 = female); ^2^ (dummy 1 = student).

**Table 6:** Main variables categorized by group avg. share.

Variables	Group 1 “The Budget-Conscious”	Group 2 “The Snack-Addicted”	Group 3 “The Health-Focused”
Gender	0.35 (0.48)	0.49 (0.50)	0.58 (0.49)
Age	28.39 (9.97)	33.18 (13.10)	29.46 (9.37)
Student	0.70 (0.46)	0.52 (0.50)	0.60 (0.49)
Snack purchase frequency ^1^	2.14 (0.77)	2.19 (0.76)	1.94 (0.87)
Purchase price snack	0.99 (0.34)	1.14 (0.38)	1.35 (0.44)
WTP REVEALED	0.83 (0.49)	1.38 (0.61)	2.40 (0.79)
Healthiness ^2^	7.85 (1.25)	7.86 (1.16)	8.24 (0.94)
Certification presence ^2^	6.88 (1.85)	6.60 (1.88)	6.78 (1.74)
Calorie content ^2^	6.17 (2.08)	6.10 (2.27)	6.43 (1.97)
Price ^2^	6.96 (1.49)	6.09 (1.32)	6.92 (1.34)
Brand ^2^	5.32 (2.04)	5.20 (2.05)	4.79 (2.13)
FB liking level ^2^	6.99 (1.51)	6.95 (1.51)	7.05 (1.66)
WTPinfoAMYLOSE	0.32 (0.55)	0.33 (0.58)	0.48 (0.58)

^1^ Data are expressed as average scores on scale 1–4 (1: very rarely–4: very often). ^2^ Data are expressed as average scores on Likert scale 1–9 (1: not at all–9: very often).

## Data Availability

The original contributions presented in the study are included in the article, further inquiries can be directed to the corresponding author.

## List of Abbreviations

FF	Functional Foods
WTP	Willingness To Pay
NC	Nutrition Claims
HC	Health Claims
NCDs	Non-Communicable Diseases
FB	Functional Biscuit
CB	Conventional Biscuit
CATA	Check-All-That-Apply Technique
RATA	Rate-All-That-Apply
UTS-test	Unpaired Two-Sample T-Tests
FMM	Finite Mixture Model
OLS	Ordinary Least Squares
WTP-R	WTP REVEALED
WTPiA	WTPinfoAMYLOSE
